# Clinicians’ Perspectives on Healthcare Cybersecurity and Cyber Threats

**DOI:** 10.7759/cureus.47026

**Published:** 2023-10-14

**Authors:** Abdullah T Alanazi

**Affiliations:** 1 College of Public Health and Health Informatics, King Saud bin Abdulaziz University for Health Sciences, Riyadh, SAU; 2 Bioinformatics, King Abdullah International Medical Research Center (KAIMRC), Riyadh, SAU

**Keywords:** health cyber attack, security measures, data breach, cyberthreats, cybersecurity

## Abstract

Introduction: In today's world, healthcare systems face various risks, including data breaches, theft, and damage. This is where cybersecurity comes in, as it helps protect sensitive personal and financial data, such as electronic health records. This study delved into the perspectives of clinicians on cybersecurity in healthcare, exploring how it impacts patient safety and the functioning of organizations. The study also identified challenges associated with implementing cybersecurity measures and the risks of not doing so.

Method: This is a qualitative study in which clinical informaticians from different health science backgrounds were asked to share their opinions using the Delphi technique, with 48 participants engaging in all three rounds.

Results: The study highlighted that 96% of participants deemed cybersecurity in healthcare critical for protecting data. Compliance with regulations (91.7%), reduced disruptions (69%), improved patient care (65%), trust (58.3%), and reputation (54%) were additional advantages. However, the study also identified top challenges to cybersecurity implementation, such as time/resource constraints (65%) and disruption to workflows/services (60.4%). Staff resistance, insider threats, and legacy system issues were also anticipated obstacles. Neglecting to implement cybersecurity measures in healthcare could lead to a higher risk of data breaches (96%), financial/legal penalties for hospitals (79%), and concerns about patient safety (65%).

Conclusion: It is imperative to prioritize cybersecurity in the healthcare industry to mitigate these risks and ensure patient confidence, health system stability, and, ultimately, save lives. A unified approach is required to enforce policies, modify behaviors, and adopt innovative practices to combat cyberattacks effectively.

## Introduction

In today's digital age, healthcare organizations face a growing threat of cyberattacks, making cybersecurity an essential aspect of healthcare management. Cybersecurity in healthcare refers to safeguarding electronic information and digital assets from unauthorized access, use, and disclosure. This includes securing sensitive patient data, medical records, and personal information from potential hackers, cybercriminals, and malicious actors. There are three primary goals of cybersecurity in healthcare: confidentiality, integrity, and availability, commonly referred to as the "CIA triad." Confidentiality pertains to keeping sensitive information private and protecting it from unauthorized access. Integrity involves ensuring that the information is accurate and has not been tampered with or altered. Availability refers to the accessibility of information to authorized users, ensuring that the information is available when needed.

Protecting information technology systems and connections from unauthorized access, data breaches, theft, damage, or manipulation of hardware or software is known as cybersecurity. It is crucial to prioritize cybersecurity when dealing with information systems that hold valuable data. With the increasing use of information technology in healthcare, enormous amounts of data are managed through these systems. Hospital information systems, especially electronic health records (EHRs), contain health-related data and personal and financial information [1].

Access to patient records from multiple sources is crucial for providing coordinated care. Improved patient outcomes and efficient resource utilization are essential for value-based care and efficiency initiatives. Informed and evidence-based decision-making by care providers and hospital executives is crucial for achieving these goals, with data serving as the core of this process. However, exchanging data across hospitals, regulatory bodies, and third-party payers increases the risk of data mismanagement, infringing on patients' privacy and potentially harming them. That is why cybersecurity is of utmost importance in healthcare [2].

The healthcare industry has been experiencing increased cyber threats and attacks, resulting in more data breaches. In the last decade, these incidents have tripled in the US, with ransomware attacks alone affecting over 42 million patients from 2016 to 2021. Almost all hospitals (90%) have had at least one data breach, with 45% experiencing five or more in 2016 [3]. Healthcare was the target of 24% of all cyberattacks in 2019. Due to these breaches, the US healthcare system is projected to incur a loss of approximately $6 billion, estimated at $7.13 million per incident, compared to $3.86 million in other industries [4-6].

Jiang and his colleagues have classified the various types of data breaches in the healthcare industry. They found that 42% were caused by theft, which included devices or personal health information. Additionally, 25% of breaches were due to unauthorized access by insiders, 21% were from hacking, and the remaining breaches occurred due to improper disposal [7]. Another study relied on self-reported surveys and revealed that only 50% of hospitals could handle cyber threats. Furthermore, hospitals have experienced a 300% surge in attacks over the past three years [8]. Medical devices are at risk of being controlled or misdirected and vulnerable to data breaches [9]. The FDA has identified 11 vulnerabilities that could impact over 200 million healthcare devices [10]. For example, a hospital experienced an incident where insulin pumps were controlled remotely, and the FDA recalled half a million pacemakers due to high vulnerability [11]. The reason for conducting this research is to gain a better understanding of how digital transformation in healthcare can affect patients and healthcare organizations, including any negative consequences that may arise from this transformation. Specifically, we explored the clinicians' perspectives on the roles and importance of cybersecurity. Our main objectives are to understand clinicians' perspectives on the importance of cybersecurity in healthcare, discover any difficulties they encounter when implementing cybersecurity measures, and assess how cyberattacks impact patients' safety and healthcare organizations' functioning. Furthermore, we aim to investigate clinicians' viewpoints on the significance of maintaining patient privacy and data confidentiality.

## Materials and methods

This is a qualitative study in which a Delphi technique was utilized to collect the opinions of clinical informaticians about cybersecurity and the risks of cyber threats to healthcare organizations. This technique aims to set a structure for communication between the expert's service providers and generate a set of priorities for healthcare organizations regarding dealing with cyber threats and the importance of setting measures to ensure cybersecurity in health organizations. The moderator has selected participants with clinical backgrounds and an interest in health information technology. Thus, the participants were selected purposively, and three rounds were held to collect responses and measure the agreement levels of the generated ideas. After each round, the moderator reviewed the information and provided controlled feedback on the collective opinions. Since no specific number of participants is recommended in the Delphi technique, the target number was set to include 10 participants for each area of interest. We targeted participants from different health sciences backgrounds: physicians, dentists, nurses, pharmacists, and allied health professionals. The aim was to recruit 60 participants to ensure a complete and accurate reflection of the opinions of different clinicians and minimize biases and inaccurate conclusions. The moderator invited potential candidates to participate in the study. Our invitation is exclusive to candidates confident in their expertise and experience in information technology, particularly in the healthcare industry. We required a candidate with a successful history of implementing and utilizing IT solutions. Other candidates were excluded from consideration. Informed consent was obtained, and participants were asked about their familiarity with cybersecurity and the cyber threats faced by healthcare organizations; furthermore, responses were solicited about the importance of cybersecurity measures and the challenges facing organizations when implementing such measures.

The study ensured ethical standards, and an institutional review board approval was obtained from King Abdullah International Medical Research Center (KAIMRC) before collecting data. Participants and individual data were kept confidential, and only aggregated data were presented.

## Results

The Delphi technique was employed to gather the viewpoints of clinical informaticians regarding cybersecurity and the threats posed by cyber-attacks to healthcare organizations. A series of three meetings was conducted for this purpose. Through this methodology, experts in the field were able to provide valuable insights into the risks and potential consequences of cyber threats. The moderator invited seventy-two participants, of which fifty-two agreed to participate. However, only forty-eight participants took part in the three rounds. The sociodemographic data of the participants are presented in Table 1. Fifty-six percent of the participants are female, and most are younger than 40 years (81%). The specialties of participants span different domains: physicians (14.6%), dentists (16.7%), nurses (18.75%), pharmacists (18.75%), and others (31%). Only 23% of the participants have a clinical experience of five years or less, while the remaining have more than five years of experience. Regarding their experience with EHRs, most participants ranked their experience as intermediate or expert (90%). Similarly, 90% of the participants feel comfortable with EHRs (moderate or high level). 

**Table 1:** The demographic data of the participants

Variable	Response	Frequency n (%)
Sex	Male	21(43.75%)
	Female	27 (56.25%)
Age (yr.)	20-29	17 (35.4%)
	30-39	22 (45.8%)
	40- older	9 (18.75%)
Specialties	Physicians	7 (14.6%)
	Dentists	8 (16.7%)
	Nurses	9 (18.75%)
	Pharmacists	9 (18.75%)
	Laboratory specialists	5 (10.4%)
	Radiology specialists	5 (10.4%)
	Other	5 (10.4%)
Clinical experience (yr.)	Five or less	11 (22.9%)
	6- 10	17 (35.4%)
	11-15	12 (25%)
	Over 15	8 (16.7%)
EHR experience (yr.)	Novice <3	5 (10.4%)
	Intermediate 3-5	16 (33.3%)
	Expert >5	27 (56.3%)
Comfort levels with EHR	Low	4 (8.33%)
	Moderate	28 (58.3%)
	High	16 (33.3%)

The advantages and importance of implementing cybersecurity measures in the short term are to protect data and preventing data breaches is the top advantage of cybersecurity in healthcare and is mentioned by 96% of the participants, followed by compliance with regulations (91.7%) and reduced disruptions in operations (69%). Other advantages include improving patient care (65%), enhancing patient trust (58.3%), and enhancing the organization’s reputation, as 54% of the participants mentioned. For long-term advantages of cybersecurity in healthcare, the participants mentioned efficient response to data breaches (89.6%), increase in healthcare efficiency (66.7%), enhanced interoperability (50%), improved reputation (37.5%), and to help to increase research and innovation (23%). Table 2 describes the short- and long-term advantages of cybersecurity in healthcare. We noticed that 63% of female participants appreciated the role of cybersecurity in improving patient trust in the short-term more than males (52%), while the role of cybersecurity, in the long-term, was appreciated in increasing healthcare efficiency by 85.7% of male participants compared to 51.9% of female participants, However, these discrepancies do not necessarily constitute a statistically significant difference as no possible test to confirm discrepancy in valuing the role of cybersecurity in improving patient trust and increasing in healthcare efficiency, based on gender.

**Table 2:** The advantages of cybersecurity in healthcare

Opportunity of Cybersecurity and Healthcare			n (%)
Short term	1	Protecting data and prevention data breaches	46 (96%)
	2	Compliance with regulations	44 (91.7%)
	3	Reduced disruptions in operations	33 (68.8%)
	4	Improved patient care	31 (64.6%)
	5	Improved patient trust	28 (58.3%)
	6	Enhanced reputation	26 (54.2%)
Long term	1	Efficient response to data breaches	43 (89.6%)
	2	Increase in healthcare efficiency	32 (66.7%)
	3	Enhanced Interoperability (connected devices)	2 (50%)
	4	Improve reputation	18 (37.5%)
	5	Help to increase research and innovation	11 (23%)
	6	Help to improve national security by protecting critical infrastructure	7 (14.6%)

The second aspect of the study is to assess and acknowledge the challenges facing healthcare organizations when implementing cybersecurity measures. Table 3 outlines the challenges of implementing cybersecurity measures in both short term and long term. Sixty-five percent of the participants mentioned implementation time, resource constraints, and disruption to workflows and services (60.4%) as the top challenges. In comparison, 44% of the participants anticipated staff resistance, insider threats (44%), and legacy system issues (35%) as challenges in the short-term cybersecurity implementation journey, while for long-term challenges, the proliferation and complexity of health information systems (75%), insider threats (68.8%), the trade-off of security, and the need to access data (40%), as the top challenges of the cybersecurity journey.

**Table 3:** The challenges of implementing cybersecurity measures in both short term and long term.

Challenges of implementing Cybersecurity and Healthcare			n (%)
Short term	1	Implementation time and resource constraints	31 (64.6%)
	2	Disruption to workflows and services	29 (60.4%)
	3	Staff Resistance and Insider threats	21 (43.8%)
	4	Legacy system issues	17 (35.4%)
	5	False Sense of Security	15 (31.3%)
Long term	1	Complexity of Systems	36 (75%)
	2	Insider threats	33 (68.8%)
	3	Balancing Security and Access	19 (39.6%)
	4	Increased reliance on technology	12 (25%)
	5	Vulnerable to supply chain attacks	5 (10.4%)

The third aspect of the study asked about the threat of not implementing cybersecurity in healthcare. The main threats and missing opportunities are described in Table 4. 96% of participants anticipated increased risk of data breaches, financial/legal penalties for hospitals (79%), and patient safety concerns (65%) without cybersecurity measures. Table 4 illustrates the answers of the participants on the expected threats of not implementing cybersecurity measures in healthcare. 

**Table 4:** Threats of not implementing cybersecurity in healthcare.

Threats of not implementing cybersecurity in healthcare			n (%)
Short term	1	Increased risk of data breaches	46 (95.8%)
	2	Financial and legal Penalties	38 (79.2%)
	3	Patient safety concern	31 (64.6%)
	4	Damage to patient trust and reputation	29 (60.4%)
	5	Loss of productivity	17 (35.4%)
Long term	1	Persistent and large-scale cyber-attacks	44 (91.7%)
	2	Legal and regulatory risks	43 (89.6%)
	3	Compromised patient safety	31 (64.6%)
	4	Compromised value-based model	13 (27%)
	5	Limited Innovation	9 (18.8%)

## Discussion

Cyber threats endanger data management in all industries, including the healthcare industry. Intruders or hackers can jeopardize the quality of data. Hence, the decisions based on these data, including compromising data quality, masking data, data loss, and exfiltration, pose an extra challenge to the healthcare industry, as they can involve stealing sensitive health information or launching ransomware attacks on hospitals [3]. The proliferating number of information technology, the need to connect devices within hospitals and outside with patients and other organizations, and the increased number of cyber threats have necessitated investigating the cybersecurity aspects and implementation in healthcare [8,12].

The risks of cyber-attacks are exacerbated in healthcare, as they can significantly reduce patient trust, disrupt health systems, and even endanger human life [13]. Therefore, it is crucial to prioritize cybersecurity in today’s healthcare organizations. Such a move would require introducing new policies and changes in human behavior, technology, and processes that must be involved comprehensively and holistically [13]. Innovation and new practices in dealing with cyber-attacks must be explored and investigated by service providers and service recipients. Onwuzuruike noted inadequate security measures in healthcare, and the current practices indicate improper security culture across health organizations [14]. The current study is among very few studies to assess the concept of cybersecurity and the risk of cyber threats from a clinician’s point of view. Additionally, the current study has recruited clinicians with a stake in health data management, believing that they would provide in-depth and more meaningful insights as they provide data to other healthcare providers in addition to their clinical duties.

In the first aspect of the study, the study assessed the participants' perspectives on the importance of cybersecurity in healthcare. The participants acknowledged and felt the urgency of implementing cybersecurity measures in healthcare, as most, if not all, of the participants prioritize the impacts of cybersecurity in healthcare. One of the main advantages of cybersecurity is protecting sensitive data, including patients' personal health information (PHI) and financial data, as indicated by 96% of the participants. Goutam indicated that various cyber threats exist and risk personal, financial, and organizational data across industries [15]. By 2025, it is estimated that the global cost of personal data theft and data loss will be $10 trillion annually, with organizations facing losses of up to $2.5 million [16]. Therefore, protecting patient data and other data should be the top driver for implementing cybersecurity measures in healthcare organizations, to ensure that systems and data are available promptly, allowing smooth healthcare operations.

Compliance with regulations is the second most crucial driver for cybersecurity and was mentioned by 92% of the participants. In the healthcare industry, multiple regulatory bodies oversee and monitor various aspects of health data management [17]. However, these bodies mandate that organizations implement broad measures to ensure compliance and avoid costly financial and legal consequences of cyber-attacks. Although the HIPAA and HITECH Act regulate and protect personal health data in the US, no specific rules are imposed on health organizations to protect data, leaving interpretation of the rules unspecified. Apart from this challenge, healthcare organizations face human and financial capital constraints, as there is a shortage of cybersecurity professionals who understand the health data context and the budgetary constraints facing most healthcare organizations [17].

Reducing disruptions in operations and improving patient care are the third and fourth drivers mentioned by the study’s participants (69% and 65%, respectively). Masking the data and losing helpful data can compromise the ability of healthcare organizations to operate and hence impact the care process. There are many incidents in which hackers have encrypted the hospital’s data and asked for payment to unlock the data. These ransom attacks are expected to grow, and hospitals are endangered for crippling their ability to operate and care for their patients [18].

The following drivers for cybersecurity are gaining patient trust and enhancing the hospital's reputation, and these drivers are mentioned by half of the participants. Hospitals have to gain patient trust by ensuring that their data are protected, and active and proactive measures are in place to prevent cyberattacks and disruptions in their daily operations [13]. Losing trust can make patients hesitant to share their data with hospitals and care providers, even clinically significant data [11]. A good reputation is crucial for any healthcare organization, as data breaches can lead to negative media coverage and damage the organization's reputation. By implementing effective cybersecurity measures and preventing data breaches, healthcare organizations can ensure their reputations remain positive [19].

As mentioned by the participants, the benefits of cybersecurity measures, in the short term, include the safety of patients' data, meeting regulatory standards, avoiding disruption and negative impact on patient care, gaining patients' trust, and maintaining a good reputation.

Healthcare organizations need to consistently maintain a high level of security as cyber threats continue to evolve. From a long-term perspective, this would ensure an efficient response to data breaches and better protection of healthcare systems and personal data. This can aid in swiftly identifying and responding to such incidents, thereby minimizing the potential risks and expenses associated with data breaches. By implementing cybersecurity measures, healthcare operations can become more efficient through automated processes, streamline data consolidation, simplify operations, and enhance healthcare service quality. Furthermore, the current and coming practice would necessitate connecting devices, and the rise of virtual care and telemedicine has led to a higher reliance on remote monitoring and other connected devices. To ensure improved healthcare accessibility and efficiency, healthcare organizations must integrate cybersecurity measures into their systems to securely utilize these devices while maintaining optimal data management standards. Another advantage of having secure data management is the potential to conduct research and develop predictive analytics and real-time data monitoring [20]. This helps to identify patterns that can lead to improved patient outcomes while ensuring the safety and confidentiality of patient data. Furthermore, by having hospital-level cybersecurity measure, it would improve national security by protecting critical infrastructure.

It is evident that introducing cybersecurity measures in healthcare has advantages. However, it can create short-term risks like resource constraints. Sixty-five percent of participants mentioned this challenge, as it can make cybersecurity measures inefficient and frustrate implementation. Expert professionals, software, hardware, and other infrastructure investments may be necessary, which can increase operational expenses [21,22]. The next challenge mentioned by 60% of the participants is the possibility of service interruptions or encountering delays. Implementing cybersecurity measures may modify the systems and require installing software and new procedures. These modifications may distribute the care processes and services, impacting patient satisfaction and revenue of the hospitals [23]. The participants mentioned that staff could be a source of struggle when implementing and maintaining data security. Insiders pose the greatest risk even to the most secure systems. The threats could be accidental, such as honest mistakes, being the victim of phishing, or intentional, in which a malicious loss or data theft [24]. Insider threats will likely increase as users become more familiar with the systems, providing more opportunities to misuse their access. Individuals with malicious intentions who have access to the system could cause severe harm to the organization, especially if there is no audit or access track.

Despite offering proper training, staff members may not possess a sufficient desire to comply with the cybersecurity measures, which could result in data breaches caused by negligence or misuse of cybersecurity tools. One of the challenges mentioned by 35% of the participants and faced by most healthcare organizations is the presence of legacy systems. These systems can be a source of threat. These systems can be obsoleted with no support from the vendor; thus, they are often more vulnerable to hacking [25]. 31% of the participants mentioned the risk of false feelings of security and cybersecurity exhaustion as a challenge to implement proper cybersecurity measures. With time, staff may become less cautious about their practice with data assuming that the system is protected and can prevent negligence. Furthermore, repeated updates and alerts about possible threats could lead to security fatigue, in which the staff ignores risks or develop resistance to new cybersecurity measures [26]. Seventy-five percent of the participants revealed that systems are becoming more complex for long-term challenges. Thus, it challenges staff members to navigate, resulting in errors and vulnerabilities, ultimately leading to breaches [27].

As cybersecurity threats evolve, healthcare organizations must stay ahead and be vigilant by developing more sophisticated security measures. It is essential to comply with regulatory standards, which mandate external audits and certifications, and to adjust to new requirements effectively. Additionally, it is crucial to regularly review and update these measures, safeguard sensitive information, and ensure that staff is always watchful in safeguarding and handling data.

The third aspect of the study is that the participants revealed that failing to implement proper cybersecurity measures may increase the risk of data breaches and financial and legal penalties (96%, and 79%, respectively). A security breach in healthcare can have various negative impacts, such as reduced productivity, financial loss, and harming the reputation of these organizations, ultimately leading to financial and legal consequences and operational inefficiencies. Neglecting cybersecurity measures in healthcare in the short term may lead to numerous risks, affecting patient safety and the entire business. Taking appropriate cybersecurity measures will enhance patient trust and bolster the financial stability of the healthcare system. While for a long-term perspective, these risks include continuous attacks, massive cyberattacks, data exploitation, financial instability, and regulatory consequences. Healthcare providers must prioritize cybersecurity and take appropriate security measures to safeguard patient data and ensure that medical services remain accessible during critical times [28].

Recommendations

The adoption of cybersecurity in healthcare proffers a myriad of advantages and is the fortified safeguarding of confidential data. Healthcare organizations are custodians of critical data, encompassing PHI and financial data. By implementing robust cybersecurity measures, organizations can reduce the probability of data breaches and the attendant potential legal and financial repercussions.

Healthcare organizations are obligated to adhere to regulatory standards that necessitate external audits and certifications. Compliance standards are subject to fluctuations; thus, cybersecurity measures may require considerable investments to accommodate new requirements effectively. Moreover, by thwarting cyber threats, healthcare organizations can bypass disruptions in their routine operations, thus minimizing healthcare delivery downtime.

The execution of cybersecurity measures within healthcare organizations presents certain hurdles that must be addressed. A primary challenge encompasses the constraints of time and resources. Given healthcare organizations' often restrictive budgets and temporal limitations, expedited implementation of crucial alterations may prove difficult. This could potentially engender an inefficient process that generates frustration for all stakeholders. In addition to allocating the necessary resources, healthcare organizations must cultivate a safety culture and cybersecurity measures awareness. Training and mandatory periodical workshops and seminars must be introduced and mandating staff to attend and participate in creating a healthy environment to protect health data and mitigate the risk of insider threats. The healthcare industry faces unprecedented risks, some of which are unanticipated and go beyond current regulations. Thus, establishing a proactive organizational framework and assessing the possible cyber threats is a vital task that organizations need to perform, considering that a given organization is as strong as the weakest link in the organization. 

Study limitations

The Delphi technique is a widely utilized research method that involves gathering expert opinions to inform emerging technologies' development and potential applications. This approach typically involves questionnaires, anonymity, and feedback to facilitate consensus among participants possessing relevant expertise [29]. However, it is essential to note that the technique's reliability, validity, and credibility have sometimes been questioned due to the subjective nature of the criteria used and the potential for a less-than-optimal outcome [30]. The approach used in this study was justified by its exploratory nature. However, future studies should focus on specific areas in cybersecurity, such as analyzing gaps in current policies and procedures, examining user behavior when handling data, identifying risks posed by insiders, anticipating potential risks in health information exchange networks, the Internet of Things, and ensuring secure connections for patients and providers outside of hospital premises or when using personal devices. 

## Conclusions

Cybersecurity is critical in healthcare to protect patient trust, prevent system disruption, and avoid endangering human life. However, limited time and resources can lead to inefficient processes, causing frustration for stakeholders. Healthcare organizations should cultivate a safety culture and cybersecurity awareness by providing training, workshops, and seminars. Establishing a proactive organizational framework and assessing possible cyber threats is crucial for protecting health data and mitigating the risk of insider threats, while addressing regulatory compliance, resource allocation, and cybersecurity awareness.
